# Dietary recommendations to customize canteen menus according to the nutritional and sensory needs of individuals with autism spectrum disorder

**DOI:** 10.1007/s40519-023-01590-z

**Published:** 2023-08-01

**Authors:** Maria Vittoria Conti, Chiara Breda, Sara Basilico, Alessia Luzzi, Luana Voto, Sara Santero, Giorgia De Filippo, Hellas Cena

**Affiliations:** 1 Laboratory of Dietetics and Clinical Nutrition, Department of Public Health, Experimental and Forensic Medicine, University of Pavia, Via Bassi 21, 27100 Pavia, Italy; 2 Clinical Nutrition and Dietetics Service, Unit of Internal Medicine and Endocrinology, ICS Maugeri IRCCS, 27100 Pavia, Italy; 3 Post Graduate Course in Food Science and Human Nutrition, Università Statale di Milano, 20122 Milan, Italy; 4 Department of Translational Medical Science, University of Naples Federico II, Via Sergio Pansini 5, 80131 Naples, Italy; 5 ImmunoNutritionLab at the CEINGE-Biotecnologie Avanzate s.c.ar.l Research Center, University of Naples Federico II, 80131 Naples, Italy

**Keywords:** Autism spectrum disorder, Collective catering, Food selectivity, Nutritional needs, Nutritional recommendations

## Abstract

Individuals with autism spectrum disorders (ASD) are often characterized by food-selectivity, food-neophobia and a marked preference for mild flavor, semi-liquid foods with pale colors. Therefore, they adopt a monotonous dietary pattern, and they prefer ultra-processed food, leading to a high risk of developing malnutrition. In Italy, where 75,072 individuals are diagnosed with ASD, center-based services play a crucial role in their daily management. Despite the centrality of nutrition in maintaining a good state of health, even more for vulnerable subjects, no validated protocol at collective catering level has been developed yet. The manuscript presents customized dietary recommendations aimed at managing the meals for individuals with ASD at collective catering service, derived from a non-systematic literature review exploring food behaviors and nutritional needs in individuals with ASD. Simple practical tips for mealtimes, such as eating together, proper seating, lighting, smell control, presenting food in a simple manner and using the same type of tableware at each meal, to meet the needs of individuals with ASD, were described. The proposal could represent a starting point in developing official guidelines aimed at ASD individuals, in collective catering service.

Level of Evidence: Level V.

## Background

In the last few years, the scientific community addressed particular attention to the investigation and description of individuals with autism spectrum disorders (ASD) behaviors [1, 2]. The scientific results highlighted which specific rituals associated with meals are adopted by the population with ASD [1–5]. These food choices are often related to the ability to integrate, to process and to modulate sensory signals (sight, touch, smell, taste) and/or oral-motor (chewing, swallowing) alteration [1, 6–9]. Individuals with ASD prefer foods characterized by soft or semi-liquid texture, pale colors, delicate tastes instead of bitterness, sourness, and spicy flavors. At the same time, they do not appreciate strong smells and high temperatures and, for all the reasons just listed, they even tend to eliminate entire food groups [1].

In addition, people with ASD show a marked preference for junk foods, with high energy density, high quantity of simple sugars, saturated fats, salt and with a low minerals and vitamins content [1, 10].

As described above, these behaviors expose individuals with ASD to a greater risk of developing overweight/obesity condition and/or nutritional deficiencies resulting in further health deterioration [1, 11–15].

Studies reported a higher prevalence of overweight (19% compared to 16%) and obesity (30.4% compared with 23.6%) between individuals with ASD and neurotypical ones [16].

Many studies described an increased risk of developing a deficiency of vitamins such as A, C, D and minerals such as zinc and iron and omega 3 in this population [16–21]. Moreover, the quality of life and the typical symptoms of these individuals are often undermined and aggravated both in the short and long term by the co-presence of gastro-intestinal dysfunctions (constipation, diarrhea, gastro-esophageal reflux), sleep disorders and sedentary lifestyle [1, 11, 22].

In the light of these considerations, it is important to underline the need to develop specific and personalized nutritional intervention programs [1, 5, 9, 12, 22–26] engaging both families or caregivers and shelters [18, 27, 28].

In this adapted setting, including a canteen service, there is paradoxically the absence of a menu meeting the needs of population with ASD [1, 5–7, 9]. The consequence of this gap is a high risk of food rejection by these individuals. Besides, the setting in which the meal takes place (lighting, noise, environment) needs to be considered as well as plating and serving (seat, plates, cutlery), since they play a central role in meal acceptance [6, 7, 29].

Shelters in charge of individuals with ASD open a potential "window of intervention", which, if correctly exploited, may allow an improvement in the nutritional status of this population throughout specifically formulated menus, resulting in a cascade of beneficial effects on their health.

The involvement of caregivers through information dossiers and nutrition education activities, such as *parent-training* and *modeling*, is also fundamental. This dual intervention approach (engaging both the shelter and the family/caregivers) will potentially lead to a positive impact on dietary habits and lifestyle of individuals with ASD.

Thus far, dietary recommendations to customize canteen menus aimed at individuals with ASD has not yet been developed, despite the European guidelines (*ESCAP guidelines*) [30], the *position stand* of the World Health Organization [31] and the most authoritative organizations in the sector (*Autism Speaks, National Autistic Society* and *AASPIRE*), underline this need.

With this manuscript, describing the relationship with food of individuals with ASD, the authors provide customized dietary recommendations for developing collective catering’ menus which could meet nutritional and sensory needs of individuals with ASD.

This could enhance food acceptability in this vulnerable population and to reduce food waste [32, 33].

### Autism spectrum disorder—clinical setting

ASD is a heterogeneous cluster of early-onset neurodevelopmental disorders that share a common symptomatology core, differing in symptom severity and intensity [34]. In the last two decades, the prevalence of ASD globally has been increasing significantly, linked both to changes in the diagnostic criteria and to increased scientific evidence and awareness of the disorder among the general population [2].

In the USA, according to data from the National Health Interview Survey (NHIS) collected in the 3-year period 2014–2016, the prevalence of ASD is 2.5% in children and adolescents [35], while in Europe it is in the range between 1.4% of the total population [36]. In Italy the estimate prevalence is 1.15%, around 1 in 77 children (age 7–9 years), 3–4 times higher in males than in females [4, 37].

Known risk factors for ASD include genetic factors, advanced age, and a history of psychiatric disorders in the parents, pre-term delivery or low birth weight, fetal exposure to insecticides, maternal exposure to bacterial or viral infections, and psychotropic therapies during pregnancy [34].

According to the latest revision of the Diagnostic and Statistical Manual of Mental Disorders (DSM-5) [38] the diagnosis of ASD is based on deficits in two domains:

- persistent deficits in communication and social interaction in multiple contexts.
- restricted, repetitive patterns of behavior, interests, or activities.

Another key assessment criterion is the level of severity and support required, which describes the degree of impairment of the individual with ASD, divided into three different levels from “support needed” (level 1) to “very significant support required” (level 3).

In addition, approximately 75% of patients with ASD have other comorbidities, either psychiatric or neurological disorders that complicate the psycho-physical picture [2, 34].

Regarding therapy, two main types of intervention are currently used: pharmacological and non-pharmacological. Pharmacological treatment relies on various classes of drugs (e.g., antipsychotics, antidepressants, anxiolytics, cholinesterase inhibitors, etc.), which bring about an improvement in symptoms and/or comorbidities, but without resolving them [2]. It is therefore important to combine this traditional approach with complementary non-drug interventions, such as cognitive/social-behavioral and music therapy, which have been shown to improve the social interaction and verbal communication of individuals with ASD [4].

It is also crucial to emphasize the central role that a nutritional education approach has in maintaining a correct state of health in individuals with ASD, precisely considering their typical risk of incurring metabolic diseases (such as overweight, obesity) and nutritional deficiencies (as outlined above).

### ASD and relationship with food

Eating is a complex action that is based on skills and functions that are often difficult for individuals with ASD [9], who frequently present patterns of Food Selectivity (FS) [1, 5, 9, 39]. It consists of an altered relationship with food and rigidity in food choices (intake of a limited number of foods, often less than five), accompanied by a poor acceptance of new foods [40, 41]. FS is common in individuals with ASD throughout the lifespan and affects 80% of individuals starting from childhood [1, 9, 41]. Its etiology is complex and probably multifactorial and may depend on gastrointestinal disturbances, anatomical anomalies, oral-motor dysfunction, metabolic disturbances, and food allergies [40–42]. The sensory hypersensitivity can lead to the refusal and avoidance of specific foods with a negative impact on the subject's diet. For example, food may be rejected due to its texture, color, smell, or temperature [43, 44]. The mechanisms involved are many and may concern: hypersensitivity to texture (soft, gelatinous, hard, crunchy, etc.), to taste (sweet, bitter, sour, etc.), to smell (both of one's own food and of that of others), to touch (fruit with or without peel, etc.), to appearance (color, shape, presentation of the dish, etc.), to temperature and to other sensory stimuli deriving from the environment in which the meal is consumed [7–9]. Furthermore, the relationship with food is characterized by repetitive attitudes at the time of the meal such as: touching the food before putting it in the mouth if it has an accepted consistency or, in the opposite way, avoiding touching it if it is slimy or moist and/or separating it inside the same plate. Therefore, it follows that an altered processing of sensory stimuli can have repercussions on a delicate moment such as that of a meal, exacerbating the related problems, especially in individuals with behavioral difficulties.

The relationship with nutrition and the consequent behavior during food consumption is a very common problem in individuals with ASD, in fact, many feel great anxiety when mealtime approaches [29]. The reasons behind this may include food neophobia, in addition to sensory aversions [45]. Maintaining a correct state of health implies the consumption of a wide variety of foods which in ASD is difficult to accept, given the known difficulties related to the management of novelty [45]. Often, these individuals are defined as "selective eaters" with a preference only for certain types of food, with “predictable” characteristics (taste, textures and color) such as ultra-processed food [1, 46]. For example, a specific brand of packaged chips will always look the same and will always have the same flavor, while an unprocessed and unpackaged product, such as a banana, could be very ripe or, on the contrary, unripe, and therefore be “unpredictable” [8, 47, 48]. So, the need for a certain food to always be identical in appearance and/or consistency and/or taste represents one of the most critical issues in the relationship with food choices and can turn meal management considerably complex [46–48].

Behavioral rigidity, definable as a difficulty in passing from one environment to another, from one activity to another [49], plays a decisive role in their relationship with food. Factors that influence behavioral rigidity include seating, plating, layout of the tables (cutlery and glasses) and the order in which foods are presented to be consumed [8].

Therefore, it is essential to learn about, to recognize and to know how to manage these critical issues in the relationship with food by people with ASD in the context of center-based service, where these individuals spend most of their time and consume main meals, such as breakfast and/or lunch and/or dinner.

### The central role of center-based services

The complex picture described above, outlines why caregivers need support in the daily management of individuals with ASD. The center-based services are a key setting for this vulnerable population [18, 28]: just considering the Italian situation, 75.072 individuals diagnosed with ASD are welcomed in center-based services daily [18, 50].

Therefore, those settings play a central role in their life, with specific responsibility in crucial moments of the day, e.g., the mealtimes.

Despite the background well described in the literature, to date menus or dietary recommendations, aimed at collective catering service and targeted to ASD individuals do not exist [1, 5, 6, 9]. This gap could lead to a high risk of refusal or reduced intake of the foods offered to individuals with ASD and to an excessive quantity of food waste, unsustainable in the long term.

In addition, no attention is frequently paid to the environment in which the meal is consumed: the researchers emphasize the need to also focus on the seat, on the dishes and cutlery used, on the way the food is presented, on the lighting, on the noises, avoiding an overcrowded environment, all of which play a role in the acceptance of food by individuals with ASD [6, 7, 46, 51].

Up today, the current real care practices for people with ASD contrast with the clinical inclusion of individuals with ASD among patients with “special health care needs” [52] and underlines the need to accompany the subject with a path of nutritional guidance starting from the diagnosis in childhood through the whole life span [53].

## Methods

### Methodological approach to non-systematic literature review

The methodological approach of the present paper led to development of customized dietary recommendations aimed at managing the meals for adults with ASD at collective catering service through a non-systematic review [54–56] exploring the current state of the literature on food behaviors and needs in individuals with ASD.

The literature research was conducted in May 2022. Studies were identified from PubMed using “autism spectrum disorder”, “health”, “nutrition”, “food selectivity”, “malnutrition condition”, “food”, “food acceptance”, and “collective catering” as key words. Search terms included the following research questions:

- “*How do individuals with autism spectrum disorder relate to food?*”
- “*What behaviors do individuals with autism spectrum adopt toward food?*”
- “*What are the health risks faced by individuals with autism spectrum disorder with respect to food consumption?*”
- “*Major nutritional deficiencies found in individuals with autism spectrum disorder.*”
- “*Altered organ functions in individuals with autism spectrum disorder (with focus on gustatory perception).*”

Narrative and systematic reviews, meta-analyses, clinical trials, guidelines, observational studies, and clinical trials conducted in humans published between 2010 and 2022 were included in the selection process. Articles published not in English were not considered in the present paper.

### Methodological approach to create customized canteen menus

To address the lack of specific dietary recommendations for collective catering services, the authors drew on the following national dietary guidelines:

- *Ministero della Salute* (2021). *Linee Di Indirizzo Nazionale per La Ristorazione Ospedaliera, Assistenziale e Scolastica* [57]; (to define the Daily Energy and nutrients intake recommendations for adults).
- *Regione Lombardia* (2022). *Linee Guida Regionali* [58]; (to define the Daily Energy and nutrients intake recommendations for adults).
- Reference Intake Levels of Nutrients and Energy for the Italian population (LARN) [59];
- *Centro di Ricerca Alimentare e Nutrizione* (2018). *Linea Guida per una Sana alimentazione* (CREA Guidelines) [60]. Recommended Weekly consumption frequencies of food groups.
- Mediterranean dietary pattern [61].

## Results

After checking for duplicates, the title and abstracts of papers were screened for inclusion by the first author. Forty-two items matched the criteria listed above. Those manuscripts were then reviewed, analyzing full text. After an initial skimming, 18 articles were selected (Table 1). The level of evidence for the included studies was at level V, narrative review.

**Table 1:** Selected articles after a non-systematic literature revision

	Authors	Title	Journal	Publication date	Article type	Objective	Population
1	Marí-Bauset, Salvador et al. [1]	Food selectivity in autism spectrum disorders: a systematic review	J Child Neurol	2014	Systematic review	To describe the Food Selectivity in Autism Spectrum Disorders	Children and adolescent with autism spectrum disorder
2	Sharma, Samata R et al. [2]	Autism Spectrum Disorder: Classification, diagnosis and therapy	Pharmacol Ther	2018	Narrative review	Full description of autism spectrum disorder: Classification, diagnosis, and therapy	Individuals with autism spectrum disorder
3	Zickgraf, Hana F et al. [5]	Rigidity and Sensory Sensitivity: Independent Contributions to Selective Eating in Children, Adolescents, and Young Adults	J Clin Child Adolesc Psychol	2022	Educational study	The study has two broad aims. The first is to explore, in a conceptual replication across four samples differing in age and diagnosis, the relationships between specific domains of sensory sensitivity and selective eating, and for the first time, the relationship between 4cognitive rigidity and selective eating. The second broad aim is to explore group differences in the degree of selective eating and the standardized effect sizes for sensory sensitivity and cognitive rigidity between general samples of young adults and children/adolescents	Parents, children, and adolescent with autism spectrum disorder
4	Kuschner, Emily S et al. [9]	A Preliminary Study of Self-Reported Food Selectivity in Adolescents and Young Adults with Autism Spectrum Disorder	Res Autism Spectr Disord	2015	Case control, Cross-sectional study	The study focuses on self-ratings of Picky eating / food selectivity in a relatively large sample of adolescents and young adults with ASD compared to neurotypical adolescents and young adults. Secondary analyses from a broader study examining brain and behavioral functioning in ASD contributes to first steps toward examining food selectivity in older adolescents and young adults with ASD	Adolescents and young adults with autism spectrum disorder and their parents
5	Doreswamy, Shriya et al. [11]	Effects of Diet, Nutrition, and Exercise in Children with Autism and Autism Spectrum Disorder: A Literature Review	Cureus	2020	Literature Review	To review articles and arrive at collective data to help primary care physicians, pediatricians, parents, and everyone involved with the treatment of autism and ASD children to improve their lifestyle	Individual with autism spectrum disorder
6	Sharp, William G et al. [12]	The Autism Managing Eating Aversions and Limited Variety Plan vs Parent Education: A Randomized Clinical Trial	J Pediatr	2019	Randomized trial	To assess the feasibility and initial efficacy of a structured parent training program for children with autism spectrum disorder and moderate food selectivity	Parents of children with autism spectrum disorder
7	Gallardo-Carrasco, Maria Carmen et al. [18]	Serum Vitamin D, Folate and Fatty Acid Levels in Children with Autism Spectrum Disorders: A Systematic Review and Meta-Analysis	J Autism Dev Disord	2022	Systematic review and meta-analysis	To describe data on the blood levels of vitamin D, folate, or fatty acids of children diagnosed with ASD	Children with autism spectrum disorder
8	Monteiro, Manuela Albernaz et al. [23]	Autism spectrum disorder: a systematic review about nutritional interventions	Rev Paul Pediatr	2020	Systematic review	To identify and analyze the scientific evidence of nutritional interventions performed in children and adolescents with autism spectrum disorders	children and adolescents with autism spectrum disorders
9	Sharp, William G et al. [25]	The Autism MEAL Plan: a parent-training curriculum to manage eating aversions and low intake among children with autism	Autism	2014	Pilot study	To (a) describe a behaviorally based parent-training program—the Autism MEAL Plan—a curriculum specifically developed to assist caregivers to Manage Eating Aversions and Low intake among children with ASD; (b) to evaluate the feasibility of the parent-training program including program content and implementation, recruitment and retention of participants, and assessment procedure; (c) to obtain preliminary outcome data evaluating the social validity and effectiveness of this curriculum using a waitlist control design	Parents of individual with autism spectrum disorder
10	Park, Hae Jin et al. [27]	Mealtime Behaviors and Food Preferences of Students with Autism Spectrum Disorder	Foods	2020	Cross-sectional study	This study aimed to examine the mealtime behaviors and food preferences of students with ASD	Adolescent with autism spectrum disorders
11	Sharp, William G et al. [28]	Scurvy as a Sequela of Avoidant-Restrictive Food Intake Disorder in Autism: A Systematic Review	J Dev Behav Pediatr	2020	Systematic review	To document the clinical presentation of scurvy in children with autism spectrum disorder and summarize the contemporary approaches to assessment and management in this population	Children with autism spectrum disorder
12	Sharp, William G et al. [41]	Feeding problems and nutrient intake in children with autism spectrum disorders: a meta-analysis and comprehensive review of the literature	J Autism Dev Disord	2013	Meta-analysis and comprehensive review	The current review sought to (a) survey the medical, habilitative, and psychological literature to identify studies using empirical methods to investigate the feeding behaviors and/or nutritional status of children with ASD and (b) summarize the evidence based on both descriptive and meta-analytic procedures	Children with autism spectrum disorder
13	Cermak, Sharon A et al. [42]	Food selectivity and sensory sensitivity in children with autism spectrum disorders	J Am Diet Assoc	2010	Comprehensive narrative review	This article provides a comprehensive narrative review of the empirical literature over the last 25 years on food selectivity and nutritional adequacy in children with autism spectrum disorders	Individual with autism spectrum disorder
14	Boudjarane, Mohamed A et al. [43]	Perception of odors and tastes in autism spectrum disorders: A systematic review of assessments	Autism Res	2017	Systematic review	Investigating the assessments of olfaction and gustation with psychophysics methods in individuals with ASD	Individuals with autism spectrum disorder
15	Kuschner, Emily S et al. [47]	The BUFFET Program: Development of a Cognitive Behavioral Treatment for Selective Eating in Youth with Autism Spectrum Disorder	Clin Child Fam Psychol Rev	2017	Pilot trial	The intervention aims to: (1) capitalize on the growing evidence for effective cognitive behavioral treatment use with individuals with autism spectrum disorder; (2) pair these approaches with exposure-based methods commonly used in feeding treatments; (3) maintain a framework of autonomy and 7self-determination; (4) develop a manualized, outpatient, multi-family group treatment program that could eventually be evaluated via a randomized controlled trial	Multi-family group
16	Raspini, Benedetta et al. [50]	Dietary Patterns and Weight Status in Italian Preschoolers with Autism Spectrum Disorder and Typically Developing Children	Nutrients	2021	Case control, Cross-sectional study	The aim of this study is to evaluate: (i) the dietary intake of Italian preschoolers with autism spectrum disorder compared to that of typically developing peers and (ii) the impact of dietary intake on weight status while considering food selectivity	Children with autism spectrum disorder
17	Esteban-Figuerola, Patricia et al. [64]	Differences in food consumption and nutritional intake between children with autism spectrum disorders and typically developing children: A meta-analysis	Autism	2019	Systematic review and meta-analysis	To determine the overall differences in nutritional intake and food consumption between children with autism spectrum disorder and control (typical development) children, as well as determine the extent to which the nutritional intake and food consumption of autism spectrum disorder children comply with the dietary recommendations	Children with autism spectrum disorder
18	Yule, Summer et al. [67]	Nutritional Deficiency Disease Secondary to ARFID Symptoms Associated with Autism and the Broad Autism Phenotype: A Qualitative Systematic Review of Case Reports and Case Series	J Acad Nutr Diet	2021	Systematic review	The primary objective of this review was to examine the relationship between the demographics, weight statuses, dietary patterns, and nutrient deficiency diseases that characterize the most severe manifestations of avoidant/restrictive food intake disorder symptomology associated with autism or the broad autism phenotype	Individual with autism spectrum disorder

### Practical tips for mealtime

In light of the eating challenges of individuals with ASD discussed above, the dining environmental context and the way the food is presented or positioned on the plate play a key role in the acceptance of food by ASD individuals [7, 62]. For this reason, some simple practical tips should be considered.

Concerning the mealtime environment, some individuals prefer to eat alone, since the mealtime can be stressful for them. Conversely, other individuals may prefer to eat together with their family or friends, which is the best option, since seeing others eat acts as a stimulus to try new foods through imitative mechanisms [27]. In fact, sitting together at the table means sharing and creating multiple exposures to different foods, increasing the potential for future tasting [12, 25]. Therefore, it is recommended to let them first sit freely where they prefer and to gradually propose to have the meal together. Moreover, considering their behavioral rigidity, especially in a domestic environment, it is advisable to use the same table for all meals and to have family members sit in the same chairs [7].

Furthermore, individuals with ASD can adopt an incorrect sitting posture at the meal table, because of weakness in the core muscles of the stomach and back, and because of poor awareness of the spatial location of their own body. For this reason, it is important to provide support and ensure the seat is comfortable, for example placing rolled up towels around the back and hips or supplying the subject with a footrest to be placed under the table, if he does not reach the floor with his feet when sitting down (mealtime tips for autistic children with eating challenges).

Moreover, in light of the sensory problems typical of ASD, it is preferable to eat the meal in a properly lightened place, avoiding intense white/cold lights (which may be unpleasant to the subject) and preferring warm lights with attenuated shades [8, 27]. In order not to affect the flavor of the meal, it is also advisable to avoid the escape of unpleasant smells from the kitchen (especially vegetables and fish) [8].

Another worth mentioning aspect in the acceptance of food by ASD individuals, in addition to texture, color, taste, shape and temperature, is the way the food is presented [1]. In particular, it is preferable not to place too many different dishes in the same plate and it is advisable to avoid touching them, which is why the use of plates divided into compartments can be useful [1, 5]. Concerning cooking methods, it is better to avoid too raw or too cooked foods, since both result in alterations of the shape, color, taste, and texture of the dish; moreover, too hot foods, can enhance intense smells and flavors often poorly accepted by individuals with ASD [7]. Finally, regarding tableware and utensils, considering the preference of individuals with ASD for "sameness" and the dietary difficulties they face, it is preferable to use the same type of plates, glasses, and cutlery at each meal [1, 12, 25, 63].

### Dietary recommendations to customize canteen menus for individuals with ASD

Following literature results, an action to respond to ASD needs is fundamental to facilitate them at mealtimes [1, 2, 4, 5, 27] with a positive impact on health [14–16, 28, 41, 64–69].

Table 2 presents a summary of the dietary recommendations addressed to collective catering services for the management and structuring of meals specifically for individuals with ASD. In addition, with respect to each food group, both the critical issues that may arise and the respective solutions are described [57–60].

**Table 2:** Dietary recommendations to customize canteen menus for individuals with ASD

Food groups	Food subgroups	Types of foods	Consumption frequencies*	Nutrients contained	Issues for consumption by individuals with ASD	Potential solutions for consumption by individuals with ASD
Group 1 Grains, derivatives and tubers	Grains and derivatives	Bread	3 ½ servings per day of 50 g	Complex carbohydrates; Dietary fiber (predominantly insoluble type); Vitamin B complex (especially B1, B2, B5, B6); Magnesium (present in high amounts for example in millet, wheat buckwheat and quinoa); Omega-6 fatty acids (especially in corn and in wheat germ)	Pronounced flavor of whole-grain products, which could cause a reduction in their consumption; Errors in the cooking time of whole grain cereals, which can lead to a hard or sticky texture; Intense color (e.g., Ermes or Venus rice)	Alternate or mix wholemeal varieties with refined varieties to gradually accustom the subject to the taste of wholemeal products; For pasta and cereals, obtain soft textures (e.g., creams/creams/ soups/risottos made from cereals and pulses); For bread, obtain crispy textures (e.g., toasted bread or bread browned in the oven or frying pan); Prepare cereals with colors generally more easily accepted (white, beige, brown)
		Pasta, rice, other grains (corn, oats, barley, spelt, etc.)	1 ½ servings per day of 80 g			
		Bread substitutes (crackers, breadsticks, etc.)	1 serving per week of 30 g			
		Baked goods, desserts (croissant, biscuits, etc.)	2 servings per week (50 g for croissants, 30 g for biscuits)			
		Cereals	2 servings per week of 30 g			
	Tubers	Potatoes	2 servings per week of 200 g			
Group 2 Fruits And Vegetables	Fruits	Fresh fruits, preserved natural wing	2 ½ servings per day of 150 g	Dietary fiber (predominantly soluble type in fruits and insoluble type in vegetables); Simple carbohydrates (glucose, fructose, sucrose) oligosaccharides, including inulin, fructo-oligosaccharides and galacto-oligosaccharides, polysaccharides); β-carotene (present especially in apricots, melons, carrots, peppers, tomatoes, parsley, etc.); Vitamin B complex (especially B5, B6, B8, B9,); Vitamin C (present especially in citrus fruits, strawberries, kiwis, tomatoes, peppers, arugula, cabbage, etc.); Potassium (present specially in spinach, in sprouts Brussels sprouts, fennel and in artichokes); Bioactive compounds (polyphenols, etc.)	Color, especially if it is a trigger; Possible presence of a fibrous texture (e.g., unripe fruit or fennel or asparagus), requiring longer chewing times; Peel characteristics (e.g., 'hairy' peach or kiwi) and manual difficulties in peeling fruit or cutting vegetables; Presence of bitter tastes (e.g., chicory, radicchio, radishes, escarole and artichokes) or sour tastes (e.g., rhubarb or citrus fruits); Unpleasant odors produced during cooking (e.g., cauliflower or Savoy cabbage or broccoli)	Present the fruit or vegetable trying to disguise its less accepted color (intense, bright colors like red, green, etc.) Present it without the peel: in fact the peel often is the main source of the fruit's color; Process the vegetable in order to mechanically break up the fibers and to obtain a soft texture (e.g., mousse in the case of fruit or creams/creams in the case of vegetables). However, procedures that result in the total removal of the fiber content (e.g., juice extractors) are not recommended; Present fruit and vegetables in an appropriate/acceptable size (e.g., diced fruit salad without added sugar, diced vegetables); Presenting sour or bitter fruit/vegetables in small doses accompanied by other, better tolerated types
		Dried fruits	Occasional consumption			
	Vegetables	Vegetables	2 ½ servings per day (200 g for vegetables such as zucchini, carrots, peppers, etc., and 80 g for salads)			
Group 3 Meat, Fish, Eggs, And Legumes	Meat	Red meat (beef, pork, sheep, horse, game)	1 serving per week of 100 g	Proteins with high biological value; Saturated fats; Trace elements (in particularly iron, zinc and copper); Vitamin B complex (especially B12); Bioactive compounds (Coenzyme Q10, creatine, bioactive peptides, etc.)	There is no evidence from the scientific literature about problems related to the consumption of this product category by individuals with ASD	There is no need, therefore, to propose solutions for consumption
		White meat (chicken, turkey, other poultry meat, rabbit)	2 servings per week of 100 g			
		Processed and preserved meat (Cooked ham and raw, bresaola, speck, bacon, mortadella, salami, canned meat)	1 serving per week of 50 g			
	Fish	Fresh or frozen fish	2 servings per week of 150 g	Proteins with high biological value; Omega-3 fatty acids (especially sardines, herring, salmon, tuna, mackerel); Omega-6 fatty acids; Trace elements (particularly iodine, zinc and selenium); Vitamin B complex (especially B12); Vitamin D	Fish with an intense taste and/or smell ('fishy' fish), given the pronounced taste and/or smell sensitivity of individuals with ASD; Manual problems in cleaning the fish, which may make consumption complex Potential presence of small residual fishbones, which may make the consumption of fish dangerous for such individuals;	Promote fish with a delicate taste and/or smell (e.g., cod, hake, sole); Avoiding the spread of fishy odor in rooms where people consume food; Process fish properly so that it is served as boneless as possible (meatballs or fillets); Use more accepted cooking techniques, such as steaming or baking, to reduce fishy smell
		Preserved fish	1 serving per week of 50 g			
	Eggs	Eggs	3 servings per week of 50 g (n° 1 egg)	Proteins with high biological value; Saturated fats; Cholesterol; Iron; Vitamin B complex (especially B2, B8, B12); Vitamin A; Vitamin D	There is no evidence from the scientific literature about problems related to the consumption of this product category by individuals with ASD	There is no need, therefore, to propose solutions for consumption
	Legumes	Dried legumes (beans, chickpeas, lentils, etc.)	3 servings per week (50 g for dried legumes; 150 g for fresh, frozen, or canned legumes)	Proteins with high biological value; Trace elements (iron, potassium, phosphorus); Vitamin B complex (especially B1, B3, B9) Dietary fiber Omega-6 fatty acids	Frequent consumption of legumes, resulting in gastro-intestinal symptoms (flatulence and abdominal bloating and pain) when consumed; Presence sometimes of a fibrous texture due to the pulses skin, potentially not appreciated; Errors in preparation, leading to a very thick consistency (e.g., if they are blended with little liquid) o hard consistency (if they are not cooked enough); Intense color (e.g., black beans, peas or broad beans); Unpleasant odors released during cooking	Propose a gradual inclusion of pulses, starting with reduced portions; Propose peeled, mashed legumes as an alternative (this reduces the fiber content) or replace legumes with the same amount (dry weight) of legume pasta (100% legume flour); Obtaining soft textures (creams or purees of pulses or legume and cereal soups; legume-based sauces such as hummus); Getting crispy textures (e.g., browning with a little oil in the oven or frying pan); Prefer legumes with optimal color (white, beige, brown) Avoid the spread of the odor produced during cooking in rooms where people consume food
		Fresh, frozen, soaked, or canned legumes (beans, lentils, chickpeas, peas, etc.)				
Group 4 Milk And Derivates	Milk and yogurt	Milk and yogurt	3 servings per day of 125 g	Proteins with high biological value; Trace elements (calcium and Phosphorus); Vitamins (B2, B12 and A); Bioactive compounds (lactoferrin, etc.); Probiotics in yogurt	There is no evidence from the scientific literature regarding problems with the consumption of this product category by individuals with ASD About milk, it is important to bear in mind that excessive consumption of milk can lead to constipation, from which these individuals frequently suffer; it is therefore recommended to respect an appropriate frequency and quantity of consumption	There is no need, therefore, to propose solutions for consumption
	Cheese	Cheese < 25 g fat/ < 300 kcal (ricotta, mozzarella, Stracchino cheese)	2 servings per week (100 g for cheese < 25 g fat/ < 300 kcal and 50 g for cheese > 25 g of fat/ > 300 kcal)			
		Cheese > 25 g of fat/ > 300 kcal (Gorgonzola, caciotta, gruyere, parmesan, pecorino cheese)				
		Cheese grated from seasoning	2 servings per day of 5 g			
Group 5 Cooking Fats	Oils	Olive oil (Virgin and extra virgin) Other cold-pressed vegetable oils (corn, peanut, sunflowers, etc.)	3 servings per day of 10 g	Essential fatty acids, saturated fatty acids; Fat-soluble vitamins (particularly vitamin E); Bioactive compounds (polyphenols, etc.)	There is no evidence from the scientific literature about problems related to the consumption of this product category by individuals with ASD	There is no need, therefore, to propose solutions for consumption. The consumption of extra virgin olive oil is recommended
	Butter Other fats of animal origin Other fats of vegetable origin	Butter Fats of animal origin (lard, suet, cream, etc.) Fats of vegetable (margarine, cream vegetables, etc.)	occasional consumption			

The table summarizes the dietary intake levels, the consumption frequencies of foods and their reference servings for the adult population in relation to LARN [59] and the CREA Guidelines [60]. Foods are classified into five categories (1. cereals and their derivatives, tubers; 2. fruits and vegetables; 3. meat, fish, eggs, and legumes; 4. milk and its derivatives; 5. fats for seasoning) according to their nutritional composition according to CREA Guidelines [60]. *Consumption portions are established considering the average requirement of an adult Caucasian individual of 2000 kcal/day, as per the guidelines [57, 58]. In addition, with respect to each food group and its nutrient composition, both the critical issues that may arise and the respective solutions are described

The purpose is to provide a reference for collective catering operators in drafting menus that could closer meet the dietary needs of individuals with ASD.

All the dietary recommendations elaborated and listed in Table 2 are based on the Mediterranean Diet (MD) model [1], a sustainable diet model with positive effects both on environment and human health [61, 70].

Regarding dietary intake levels, as there are no specific recommendations for individuals with ASD, the authors referred to the LARN [59]; whereas, for consumption frequencies of the respective food groups, the CREA Guidelines, revision 2018 were considered [60].

In addition, the National Guidelines for Hospital, Welfare and School Catering [57] were considered for the daily Energy Intake. For the adult population, it suggests an energy intake of about 2000 kcal, divided into three main meals (breakfast 20%, lunch and dinner 40% each); possible snacks can contribute by providing 10% of the caloric share with a consequent percentage reduction of the other meals [57].

Furthermore, according to the Guidelines of the Lombardy Region for Catering [58], the macronutrient composition of lunch and dinner should be as follows: approximately 15% of total calories from protein, 30% from lipids and 55–65% from complex carbohydrates [58].

Getting into the heart of Table 2, the authors describe the recommendations for each of the five reported food groups.

Group 1: Grains, derivates and tubers. The main observed issues are the rejection of whole grain products due to their texture and flavor. To overcome this problem the authors suggested mixing together refined and whole grains and alternating the consumption of pasta with that of dishes with a creamy texture that are easy to chew (e.g., rice or barley soup, whole grain cornmeal polenta).

Group 2: Fruits and vegetables. The main observed issues are colors and fibrous textures. To overcome this problem the authors suggested presenting this fruit group according to soft colors which are more accepted. Regarding vegetables, is preferable to process them obtaining mousse or creams; regarding fruits, it is preferable to serve them peeled and cut into small pieces to facilitate swallowing.

Group 3: Meat, fish, eggs, and legumes. In reference to meat, the major issue is related to chewing. Therefore, the suggestion is to prefer cuts that are lean, tender, and not stodgy (e.g., minced meat). In reference to fish, intense odor is a major reason for rejection. Therefore, it is suggested to prefer fish with neutral odor (e.g., sea bream, sea bass, cuttlefish, or squid) and without bones. The problems most related to the consumption of legumes are like those of vegetables. Therefore, it is recommended to serve them in preparations with creamy and soft textures (e.g., creams, hummus, etc.). Also, it is preferable to present them in the form of more accepted foods (e.g., meatballs, burgers or mixed with pasta sauce).

Group 4: Milk and derivates. This food category is positively accepted. The only advice is to prefer low-fat soft cheeses with muted odors (e.g., mozzarella, ricotta, stracchino).

Group 5: Cooking fats. There are no evited problems related to the acceptance of this food group. However, it is suggested preferring extra virgin olive oil and avoid butter, margarines, and dressing (e.g., mayonnaise, BBQ sauces, etc.)

## Conclusions

As set forth in this review, the dining environment and presentation of food are critical in individuals with ASD, and, if not well managed, can lead to food refusal, reduced intake, nutritional deficiencies, and excessive food waste.

Despite this, to date no specific dietary recommendations have been developed for individuals with ASD aimed at collective catering service.

The proposed customized dietary recommendations addressed to collective catering services for the management and structuring of meals specifically for individuals with ASD could be a starting point to develop official guidelines specifically for individuals with ASD in home and shelter contexts.

## Strength and limits

The limitations of this review are related to its narrative nature. Given the great heterogeneity of the selected papers, it was not possible to produce a statistical analysis. However, the authors took a structured and methodical approach to presenting the results. Nonetheless, our data add support to the existing literature on food selectivity in individuals with ASD and put a spotlight on the need to develop interventions, particularly at the level of collective catering service, that aim to improve the eating habits of individuals with ASD resulting in a positive impact on their health, since to date there are no official guidelines.

## What is already known on this subject?

Individuals with autism spectrum disorder (ASD) frequently present patterns of food selectivity. Due to their restrictive food choices (e.g., preference for pale colors, delicate tastes, and dislike for strong smells and high temperature) they tend to eliminate entire food groups. This could lead to marked preference for foods with high energy density, high quantity of simple sugars, saturated fats, salt and with a low minerals and vitamins content. As a result, these behaviors expose individuals with ASD to a greater risk of developing overweight/obesity conditions and/or nutritional deficiencies resulting in further health deterioration.

## What this study adds?

Up-to-date menus or nutritional recommendations, aimed at collective catering service and targeted to ASD individuals do not exist. The authors provided the redaction of National nutritional recommendations for collective catering to better manage the diet of individuals with ASD which can serve as a starting point in developing official guidelines.

## Data Availability

Data are available on request from the authors.
